# A Hydrogel as a Bespoke Delivery Platform for Stromal Cell-Derived Factor-1

**DOI:** 10.3390/gels8040224

**Published:** 2022-04-06

**Authors:** Yi Wang, Vanessa Penna, Richard J. Williams, Clare L. Parish, David R. Nisbet

**Affiliations:** 1 The Graeme Clark Institute, The University of Melbourne, Melbourne 3010, Australia; yi.wang24@unimelb.edu.au; 2 Department of Biomedical Engineering, Faculty of Engineering and Information Technology, The University of Melbourne, Melbourne 3010, Australia; 3 The Florey Institute of Neuroscience and Mental Health, The University of Melbourne, Melbourne 3052, Australia; vanessa.penna@florey.edu.au (V.P.); clare.parish@florey.edu.au (C.L.P.); 4 Institute for Mental and Physical Health and Clinical Translation, School of Medicine, Deakin University, Melbourne 3216, Australia; richard.williams@deakin.edu.au; 5 Laboratory of Advanced Biomaterials, Research School of Chemistry and the John Curtin School of Medical Research, The Australian National University, Canberra 2601, Australia; 6 Melbourne Medical School, Faculty of Medicine, Dentistry and Health Science, The University of Melbourne, Melbourne 3010, Australia

**Keywords:** cell transplantation, self-assembly peptide (SAP), hydrogel, laminin, stromal-cell-derived factor (SDF), cell survival

## Abstract

The defined self-assembly of peptides (SAPs) into nanostructured bioactive hydrogels has great potential for repairing traumatic brain injuries, as they maintain a stable, homeostatic environment at an injury site, preventing further degeneration. They also present a bespoke platform to restore function via the naturalistic presentation of therapeutic proteins, such as stromal-cell-derived factor 1 (SDF-1), expressed by meningeal cells. A key challenge to the use of the SDF protein, however, is its rapid diffusion and degradation. Here, we engineered a homeostatic hydrogel produced by incorporating recombinant SDF-1 protein within a self-assembled peptide hydrogel to create a supportive milieu for transplanted cells. Our hydrogel can concomitantly deliver viable primary neural progenitor cells and sustained active SDF-1 to support the nascent graft, resulting in increased neuronal differentiation. Moreover, this homeostatic hydrogel can ensure a healthy and larger graft core without impeding neuronal fiber growth and innervation. These findings demonstrate the regenerative potential of these hydrogels to improve the integration of grafted cells to treat neural injuries and diseases.

## 1. Introduction

Cell replacement therapy has great potential for the regeneration of neural circuity arising from brain diseases and injuries. Conceptually, new cells are administered into the site of damaged tissue, where they can replace the dead cells, and facilitate repair and/or the protection of the damaged tissue [1,2]. Practically, however, there are challenges. While preclinical and clinical studies provide evidence that cell transplantation improves functional recovery and display symptomatic relief [3], the extent of these effects is dependent on the viability and integration of grafted cells. The survival of these cells, however, can be hindered by several stresses during their procurement, preparation, implantation, and integration. To address this, research efforts increasingly focused on two key factors: the modality of cell administration, and the provision of a supportive biomimetic environment during the early stages of the regenerative process [4].

Stromal-cell-derived factor 1 (SDF-1) is expressed by the meninges [5] and plays diverse roles during essential biological processes, most notably in neural development such as neurogenesis, cell migration, and angiogenesis [6,7,8,9]. In the context of neural repair, chemokine SDF-1 plays a role in both cell survival and cell adhesion [10,11]. When cotransplanted with neural progenitors, meningeal cells promoted survival and engraftment of dopamine progenitors in a mouse model of Parkinson’s disease (PD) [12] through mechanisms likely attributed to SDF-1 secretion from the meningeal cells. However, the ability to directly assess this through the controlled and sustained delivery of native SDF protein is constrained by both its rapid diffusion and degradation by exopeptidases, such as dipeptidyl peptidase-4 (DPP-4) and matrix metalloproteinase-2 (MMP-2) [13,14]. Therefore, to exploit its therapeutic effects, SDF protein must be protected and restrained to ensure local functional delivery.

Hydrogels formed by self-assembled peptides (SAPs) enable the long-term delivery of a range of biologically useful macromolecules, such as growth factors [15,16,17], engineered proteins [18], viral vectors [19,20], and chemotherapeutic drugs [21], providing a promising mechanism to ensure localised functional deliveries. Thanks to the readily engineered peptide chain that forms their backbone, SAP hydrogels were developed as an attractive biomaterial for brain repair by matching selected features of the extracellular matrix (ECM) that composes the bulk of the central nervous system (CNS) [22,23]. Previous studies showed the utility of such SAP hydrogels to support human neuronal stem cells or progenitor cells in intact [24], stroke-injured [25], and PD [16] brains. These SAP hydrogels can deliver cells, as they can be reversed from gel into liquid through shearing [26], rendering them amenable to mixing with cells prior to injection. These hydrogels can effectively fill a void at the site of brain injury [27] and sustain the presentation of functional proteins [16,17,28]. Our previous study showed that SAP hydrogels were able to present a biological active motif (isoleucine–lysine–valine–alanine–valine, IKVAV) derived from protein laminin as the major component of ECM [29]. This biological epitope promoted neural cell adhesion, survival, differentiation, and plasticity [30,31].

We hypothesised that a homeostatic hydrogel could be engineered by encapsulating SDF-1 within an SAP hydrogel, which could then be administered into the brain to create an environment that is beneficial to cell survival, differentiation, and integration. In this work, the homeostatic hydrogel provided a sustained release of SDF over 28 days. Post-transplantation, the homeostatic hydrogel was able to support graft growth, reflected by the significantly increased size of the graft core, suggesting that there was enhanced survival and/or proliferation of the implanted cells. Furthermore, the SDF-functionalised hydrogel supported neuronal maturation within the graft. Overall, these findings demonstrate the capacity of functional homeostatic hydrogels to improve both the survival and development of grafted cells for the treatment of neural injuries and diseases.

## 2. Results and Discussion

### 2.1. Results

#### 2.1.1. Homeostatic Hydrogels Enable the Sustained Release of SDF

**Figure 1:**
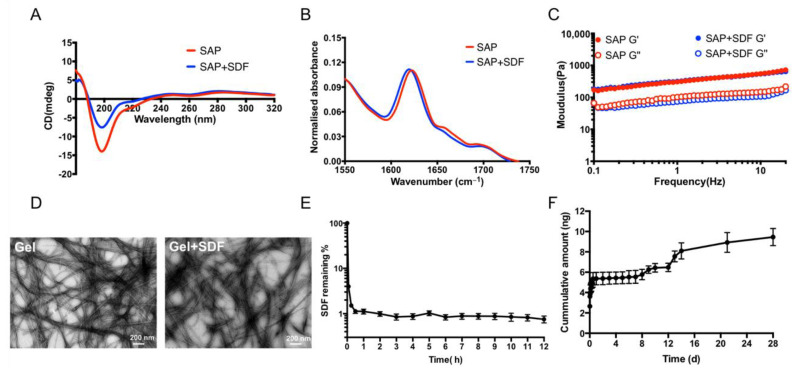
Homeostatic hydrogel maintains its characteristics and supports sustained release of SDF protein. (**A**) Characteristics of hydrogels with/without SDF via circular dichroism (CD), (**B**) Fourier transform infrared (FTIR), (**C**) rheology, (**D**) transmission electron microscopy (TEM), (**E**) recombinant SDF protein degradation in media, (**F**) cumulative release of SDF protein from SAP hydrogel. Data represent mean ± standard error of the mean (SEM).

We optimised a previously described SAP hydrogel that presents the functional epitope of laminin (IKVAV) for the in vivo delivery of cells. Laminin is the key protein within the CNS extracellular matrix that plays a role in neural adhesion, proliferation, differentiation, and plasticity [32]. In order to create the homeostatic hydrogel, SDF was incorporated via shear encapsulation. Gentle vortexing induced a gel-to-solution transition that enabled the thorough mixing of the recombinant SDF protein throughout the system. Then, upon shear cessation, the hydrogel reformed with a uniform, constrained presentation of SDF protein. The postformulation characteristics of this homeostatic hydrogel were then determined by circular dichroism (CD), Fourier transform infrared (FTIR) spectroscopy, rheology, and transmission electron microscopy (TEM) (Figure 1A–D). CD showed a transition peak between 180 and 220 nm, and FTIR displayed major peaks at 30 cm^−1^ and minor speaks at 1690–1700 cm^−1^ in the amide I region, indicating the formation of β-sheets within the fibrous network of the hydrogel. These data demonstrated that incorporating the SDF protein had no deleterious effect on the bioactive secondary structure of hydrogel. Rheology showed that the hydrogel was maintained, with storage modulus G’ dominant to loss modulus G” within the range of measured frequencies, indicating viscoelastic properties in a suitable range for neural tissue [16]. The morphological properties of hydrogel were determined by TEM, showing that the observed supramolecular ordering was associated with the formation of nanofibres.

The release profile of SDF was investigated via enzyme-linked immunosorbent assay (ELISA). First, we assessed SDF degradation in media, showing that SDF degraded rapidly within 30 min, with approximately 1% of protein remaining after this time (Figure 1E). SDF encapsulated within the homeostatic hydrogel maintained sustained release of SDF for 28 days, demonstrating the ability of homeostatic hydrogel to stabilise and deliver functional protein.

#### 2.1.2. Homeostatic Hydrogels Are Biocompatible In Vivo

**Figure 2:**
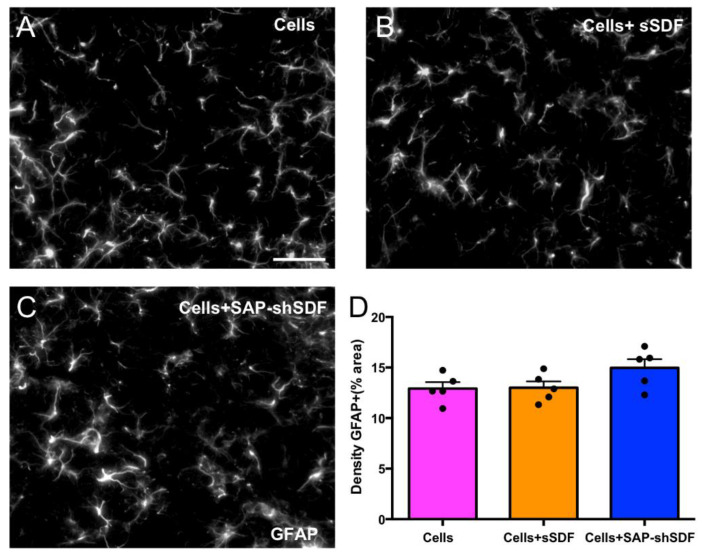
Homeostatic hydrogel has no effect on the host inflammatory response. (**A**–**C**) Representative images of GFAP+ immunolabeling adjacent to GFP+ graft in Cells, Cells + sSDF, and Cells + SAP-shSDF groups, respectively, scale bar = 50 μm; (**D**) density of GFAP+ reactive astrocytes surrounding the GFP+ graft. Data represent mean ± standard error of the mean (SEM), *p* = 0.114.

To further explore the performance of the homeostatic hydrogel in vivo, we assessed its biocompatibility. We compared the host immunological response to implanting cortical progenitors derived from fetal tissue (Cells) to cell grafts in the presence of SDF, administered either via acute bolus delivery (100 ng of recombinant SDF-1 protein together with the cells; Cells + sSDF) or sustained delivery from the SAP hydrogel (Cells + SAP-shSDF). After 8 weeks, there was no significant increase in the density of proinflammatory GFAP+ astrocytes immediately adjacent to the site of hydrogel administration observed within the brain between the homeostatic hydrogel group and other groups (Figure 2). This demonstrated that there was no immunogenic response to either the SDF protein or the homeostatic hydrogel. Data confirmed that homeostatic hydrogel was biocompatible and a strong candidate for further in vivo study.

#### 2.1.3. Homeostatic Hydrogels Support Graft Growth

**Figure 3:**
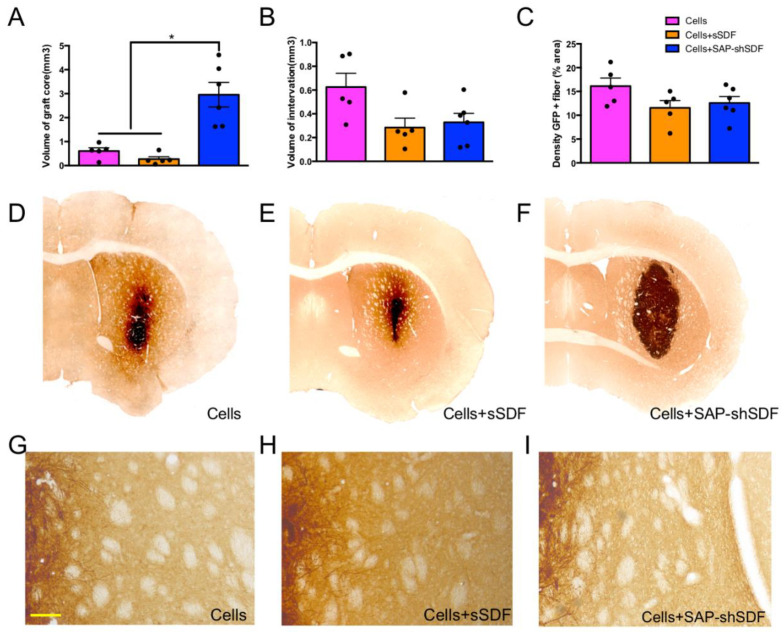
Homeostatic hydrogel supports graft growth. (**A**) volume of graft core, (**B**) volume of graft innervation within host, (**C**) quantification of GFP fiber density, (**D**–**F**) representative photomicrographs providing a coronal view of GFP+ graft and innervation of GFP+ graft in all groups, (**G**–**I**) representative images of GFP+ fiber obtained adjacent to the graft in all groups, scale bar = 100 μm. Data represent mean ± standard error of the mean (SEM; *, *p* < 0.05).

With the confirmation of biocompatibility, we next assessed the impact of acute and sustained SDF delivery on graft integration. The isolation of fetal donor tissue from green fluorescent protein (GFP+) expressing embryos enabled the clear distinction of the donor (GFP+) cells within the host (GFP−) brain. The homeostatic hydrogel (Cells + SAP-shSDF) significantly increased the volume of graft core by 4.8-fold by 8 weeks post-transplantation compared to the Cells group, and by 10.9-fold compared to the Cells + sSDF group (Figure 3A,D–F). The use of the tripartite (Cells + SAP-shSDF) homeostatic hydrogel was thus determined to be the dominant contributing factor to the increased volume of the graft core. The homeostatic hydrogel had no influence on the capacity of the cells to innervate the host tissue, with the volume of graft innervation (Figure 3B,D–F, *p* = 0.084) and density of GFP + fibers remaining unchanged across groups (Figure 3C,G–I, *p* = 0.260). These data confirmed that homeostatic hydrogel promoted the survival of the implanted progenitors, ensuring a larger graft core of similar transplanted cell density. This highlights the capacity of the homeostatic hydrogel to enhance the efficacy of cell transplantation.

#### 2.1.4. Homeostatic Hydrogels Promote Differentiation of Cell Graft

**Figure 4:**
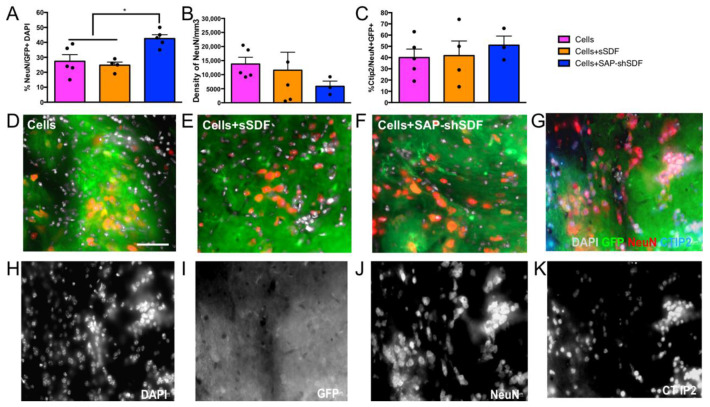
Homeostatic hydrogel shows maturation of cell grafts and neuronal specification. (**A**) Quantification of percentage of NeuN + DAPI + cells within delineated GFP+ graft, (**B**) quantification of NeuN + density within the delineated GFP+ graft, (**C**) quantification of percentage of Ctip2+ NeuN+ cells within the delineated GFP+ graft, (**D**–**F**) representative images showing NeuN + DAPI + cells within GFP+ grafts, scale bar = 50 μm, (**G**–**K**) representative images in Cells + sSDF groups expressing DAPI, GFP, NeuN, and CTIP2. Data represent mean ± standard error of mean (SEM; *, *p* < 0.05).

The goal of cell transplantation is to integrate new neurons within the host brain for the purpose of replacing lost neural circuits and/or the modulation of the host environment (for example, protection of surrounding residual host neurons). Therefore, it was necessary to assess the capacity of grafts to mature into postmitotic neurons. We assessed the proportion of the GFP+ graft derived cells that expressed postmitotic protein NeuN (i.e., NeuN + /GFP + DAPI). Results showed that there was a significantly increased proportion of identifiable, viable neurons in the graft of homeostatic hydrogel (42.5 ± 2.5%, Figure 4A,F) compared to other groups (Cells group: 27.4 ± 4.4%; Cells + sSDF group: 24.7 ± 2.1%; Figure 4A,D,E), demonstrating the maturation of the neurons. In addition, grafts were rich in βIII-tubulin + cells (Supplementary Figure S1), which are indicative of immature neurons. This result demonstrates that homeostatic hydrogel effectively supports the maturation of grafted cells, potentially providing a pool of neurons for the treatment of those brain diseases or injuries where there is loss of neural cells or neural circuity and lack of functional integration. Lastly, we assessed the capacity of neurons to mature into cortical fates, noting their origin from the developing fetal cortex. With deep layer cortical neurons (expressing CTIP2) maturing prior to upper layer cortical neuron populations, we specifically looked at the proportion of these cells within the grafts. No significant difference was observed in the proportion of CTIP2 + neurons among all groups (Figure 4C), suggesting that functionalised hydrogel, while promoting neuronal maturation, had no impact on laminar fate.

### 2.2. Discussion

There is a pressing clinical need to reproducibly increase cell survival during cell transplantation. Cell replacement therapy is an attractive treatment option for brain diseases and injuries, whereby cells are employed into therapeutic sites for repairing or replacing the neural cells of the damaged tissue with the goal is to recover or regenerate functional neural circuity providing symptomatic relief and even functional recovery [2]. The functionality of a graft is evidently dependent on its survival; hence, significant efforts are undertaken to protect and optimise implanted neural progenitors. Cell viability can be influenced by many factors, such as injection procedure [33], the lack of a physical environment [34], and insufficient trophic support [35], and is rightly an intense focus. Here, we engineered a homeostatic hydrogel that was an SAP hydrogel incorporated with SDF providing cell protection during administration, offering a biomimetic environment for cell survival and integration via the maintenance of a uniform environment and sustained SDF release during cell development.

Chemokine SDF-1 is involved in various biological processes, including neurogenesis, angiogenesis, and inflammatory processes in the CNS [36]. However, the SDF protein degraded rapidly in 30 min in media (Figure 1E), which is similar to previous studies [13,14] where an ideal sustained delivery of SDF is required. Shear loading proteins into SAP hydrogel could protect them from degradation, providing sustained and long-term release [15,16]. The cumulative release of SDF from SAP hydrogels for 28 days was achieved, which confirmed the homeostatic hydrogel enables the successful delivery SDF in a sustained manner. More importantly, the incorporation of SDF did not disturb the properties of the SAP hydrogel (Figure 1A–D). The SDF group or the homeostatic hydrogel showed in vivo biocompatibility (Figure 2) with fewer immune cells (astrocytes), similar to the Cells group. This is significant, as biocompatibility is a decisive factor for cell transplantation as it does not result in the formation of glial scars [37].

The increased volume of graft core was observed in the homeostatic hydrogel group (Figure 3A). Previous studies utilising SDF-1 did not support the proliferation of neural progenitor cells without the additional of other growth factors [38]. Thus, this increased volume of the graft core was achieved due to enhanced maturation, which was induced through Akt phosphorylation and FOXO3a activation by activating the receptor of SDF-1, CXCR4 [39]. There was no effect on graft innervation (Figure 3B,D–F) and density of GFP+ fiber (Figure 3C,G–I) across groups, which is consistent with previous findings [24]. The ability of integration of grafted cells into the host tissue is without a hindrance effect. These findings highlight that the hydrogel did not impede the plasticity of the grafted cells. We observed a high proportion of neurons in the homeostatic hydrogel group compared to in the controls (Figure 4A). This may have been due to the two parameters that were designed into the hydrogel: (i) the high density of laminin (IKVAV) epitope on the nanofibrous fibers of SAP hydrogel presents biological signals for cell adhesion, cell differentiation, and plasticity [30]; and (ii) SDF-1 directly promotes the maturation of neural progenitor cells into neurons [40]. We observed no effect on density of neurons (Figure 4B) or difference in layer specificity between groups (Figure 4C), which may have been because neither SDF nor SAP hydrogel negatively affected the development of neurons from the primary cortical neural progenitor cells.

## 3. Conclusions

We presented a homeostatic, fully synthetic hydrogel that can mimic the ECM and provide a platform to encapsulate SDF-1. This enables the naturalistic presentation of SDF-1 with sustained release of functional units over 28 days. This unique combination of microenvironment and constant presentation enhances survival, increases the number of residual neurons, and does not impinge on the maturation of grafted cells. This class of homeostatic hydrogel is a facile yet bespoke route to enable an unobtrusive medium that is easily modified to be beneficial to enhancing outcomes of cell transplantation. This material approach holds significant potential for improving not only the treatment of brain injuries and diseases, but a whole raft of tissue repair applications.

## 4. Materials and Methods

### 4.1. Solid-Phase Peptide Synthesis

N-fluorenylmethoxycarbonyl self-assembly peptide (Fmoc-SAP) was manually synthesised via solid-phase peptide synthesis (SPPS) as previously described [26]. SPPS was carried out in a rotating glass reactor vessel at a 0.4 mmol scale. The process involves the stepwise deprotection and coupling of bead resin-attached amino acids (AA) (GL Biochem, Shanghai, China). More specifically, synthesis started with deprotection in 20% piperidine in dimethylformamide (DMF, Sigma-Aldrich, St. Louis, MO, USA). Then, the following amino acid was coupled with the addition of an Fmoc–AA mixture solution including Fmoc-protected amino acid (2 mmol), O-benzotriazole-N,N,N’,N’-tetramethyl-uronium-hexafluoro-phosphate (HBTU, 1.92 mmol, 720.00 mg, Sigma-Aldrich, St. Louis, MO, USA), hydroxybenzotriazole (HoBt, 2.00 mmol, 272.00 mg, Sigma-Aldrich, St. Louis, MO, USA), N,N-diisopropylethylamine (DIPEA) (4.8 mmol, 0.80 mL, Sigma-Aldrich, St. Louis, MO, USA), and 8 mL DMF. After the final coupling step, the resin was washed with ethanol 3 times and dried under a constant vacuum oven for 48 h. A Kaiser test was used for the detection of free amines to confirm the success of each deprotection and coupling.

After drying, Fmoc-SAP was cleaved from the resin using trifluoroacetic acid (TFA, Sigma-Aldrich, St. Louis, MO, USA) with 2.5% distilled water and 2.5% triethylsilane (TES, Sigma-Aldrich, St. Louis, MO, USA) for 2 h with shaking every 30 min. Then, the solution was filtered via glass wool to remove the resin, followed by nitrogen sparge until the final volume was less than 5 mL. Dropwise hydrochloric acid (HCl, Sigma-Aldrich, St. Louis, MO, USA) or cold ether (Sigma-Aldrich, St. Louis, MO, USA) was applied to precipitate peptides 5 times, followed by drying under constant vacuum for 7 days before grinding into a fine powder using a metallic microspatula for research use.

### 4.2. Preparation of SAP Hydrogel

SAP hydrogels (DDIKVAV) were prepared as previously described; please see [26] for details regarding the hydrogel preparation and for a schematic diagram of the system. Briefly, peptide powder (Fmoc-SAP) was suspended into 100 μL of DI H_2_O with 50 μL 0.5 M sodium hydroxide (NaOH, Sigma-Aldrich, St. Louis, MO, USA). Then, the dropwise addition of 0.1 M hydrochloric acid (HCl) with continuous vortexing was monitored with pH of peptide solution change until reaching the physiological pH (around 7.4). Typically, around 100–150 µL of 0.1 M HCl was allowed. Once gel formed with the optimal pH attained, PBS was added to bring the gel up into 15 mg/mL concentration. The pH of the gels was measured with an Oakton pH 700 micro pH electrode (Thermo Fisher Scientific, Waltham, MA, USA). The resultant hydrogels could be reversed to an aqueous state by application of shear force via vortexing. After gentle vortexing into a solution state, recombinant mouse SDF-1 (R&D systems, Minneapolis, MN, USA) was loaded into SAP hydrogels. Then, upon cessation of shear force, the hydrogel reformed with a uniform, constrained presentation of the SDF protein. This shear loading of the protein during the aqueous phase of the hydrogel allowed for homogeneous distribution throughout the gel.

### 4.3. Circular Dichroism (CD)

CD was performed to determine the secondary structure of hydrogel. The hydrogel was diluted at a 1:10 ratio of hydrogel and DI H_2_O to reduce the scattering effects. Diluted peptide hydrogel around 400 µL was added into the cuvette with a 10 mm pathlength. CD scans ranged from 180 to 320 nm with a step size of 0.5 bandwidths using a Chirascan CD spectrometer (Applied Photophysics Limited, Leatherhead, UK), and a baseline (DI H_2_O) was subtracted. Resulting data were averaged and smoothed postacquisition using Chirascan software (V100).

### 4.4. Fourier Transform Infrared Spectroscopy (FTIR)

FTIR was performed to check the peptide bonds by using an Alpha Platinum Attenuated Total Reflectance (ATR) FTIR (Bruker Optics, Billerica, MA, USA). Approximately 30 μL of peptide hydrogel was placed on the single reflection diamond, absorbance scans of the amide I region (1550 to 1750 cm^−1^) were obtained for each peptide, and a background buffer scan was subtracted.

### 4.5. Rheology

Rheological analysis was performed to examine the viscoelastic properties of the formed Fmoc-SAP hydrogel by using a Kinexus Pro+ rheometer (Malvern Panalytical, Cambridge, UK). Approximately 200 μL of peptide hydrogel was placed on a 20 mm rough flat plate with solvent trap, and the geometry was placed on the top with upper rim trap (Lower Geometry: PLS55 C0177 SS, Upper Geometry: PU20 SR1351 SS). A gap size of 0.2 mm was set up, and multiple frequency sweeps were performed for frequencies in the range of 0.1–100 Hz with a 0.1% oscillatory strain at a constant temperature of 37 °C.

### 4.6. Transmission Electron Microscopy (TEM)

Negative stain transmission electron microscopy (TEM) was performed on a Hitachi H7100FA electron microscope (Hitachi, Tokyo, Japan) with a LaB6 cathode at 100 kV. Negative staining of samples was prepared as followed. Formvar-coated copper grids were glow-discharged for 30 s at 15 mA. Hydrogel samples were negatively stained with 0.75% uranyl formate (UF). After glow discharge, grids were gently immersed in the samples for 30 s, briefly immersed in two consecutive drops of Di H_2_O, and immersed into two consecutive drops of UF for 30 s. Grids were gently blotted dry between immersions.

### 4.7. Release Profiles and Enzyme-Linked Immunosorbent Assay (ELISA)

For the release profiles of SDF-1 from the gel, stromal cell-derived factor-1 (SDF-1100 ng, R&D Systems) was loaded into the SAP hydrogel (100 µL) by reverse shearing to an aqueous state and then casting into wells of a 96-well plate. The hydrogel was allowed to settle for 5 min to reform into a gel. Then, 200 µL of PBS was gently added to the well and incubated at 37 °C. The supernatant was collected at predetermined intervals over a 28-day period. Collected samples were stored at −80 °C in a low-temperature freezer until analysis.

Enzyme-linked immunosorbent assay (ELISA) was used to quantify the amount of SDF-1 released. SDF-1 levels were quantified using a Quantikine ELISA kit (R&D Systems) following the manufacturer’s instructions and our previously described methods [41]. Release profiles were performed in three wells for each time point and repeated in three independent experiments.

### 4.8. In Vivo Transplantation of Cells with SDF-1 and Tissue Processing

All animal procedures and methods were conducted in accordance with the Australian National Health and Medical Research Council’s published Code of Practice for the Use of Animals in Research, and were approved by the Florey Institute of Neuroscience and Mental Health Animal Ethics Committee. Cells for transplantation were obtained from time-mated mice expressing green fluorescent protein (GFP) under the β-actin promoter, which enabled the clear distinction of the grafted cells (GFP+) cells within the host brain. Cortical brain tissue was isolated from pups at embryonic day 13.5 (E13.5), dissociated, and resuspended at 100,000 cells/µL until the time of surgery. Cells and gels were mixed at a 1:1 ratio immediately prior to in vivo delivery. Animals were group-housed in individually ventilated cages on a 12/12 h light/dark cycle with ad libitum access to food and water. Adult swiss mice received transplants of 1 of the 3 following treatments: (i) cells (Cells group, n = 6), (ii) cells + acute delivery of SDF-1 (100 ng, Cells + sSDF group, n = 6), (iii) cells + SAP containing shear encapsulated SDF-1 (Cells + SAP-shSDF group, n = 6). Animals were anaesthetised with 2% isoflurane and placed in the stereotaxic frame. A craniotomy was performed, and unilateral microinjections of cells and hydrogels (total 2 µL) were implanted into the host striatum (0.5 mm anterior and 2 mm lateral to Bregma, and 3 mm below the surface of the brain).

After 8 weeks, mice were euthanised by an overdose of sodium pentobarbitone (100 mg/kg) and transcardially perfused with warm saline followed by 4% paraformaldehyde (PFA). Brains were removed, postfixed for 2 h in 4% PFA, and cryopreserved overnight in 30% sucrose solution. Brains were sectioned on the coronal plane using a freezing microtome (40 µm thickness, 1:12 series).

### 4.9. Immunohistochemistry

Immunohistochemistry was performed on free-floating brain sections as previously described [25]. In brief, brain sections were washed and incubated in primary antibodies overnight at room temperature. Primary antibodies and dilution factors were as follows: rabbit anti-GFAP (antiglial fibrillary acidic protein, 1:1000, Abcam, Cambridge, UK), rabbit anti-GFP (1:20,000, Abcam), chicken anti-GFP (1:1000, Abcam), mouse anti-NeuN (1:1000, Abcam), Rat anti-CTIP2 (1:500, Abcam), mouse anti-βIII-tubulin (1:2000, Promega, Madison, WI, USA), rabbit anticollagen I (1:200, Abcam). The following day sections were rinsed and blocked in 5% donkey serum for 20 min. Secondary antibodies for (i) direct detection were used at a dilution of 1:200: DyLight 488, 555, 647 conjugated donkey antirabbit, antichicken, antirabbit, antirat (Jackson ImmunoResearch Laboratory, West Grove, PA, USA); and (ii) indirect with streptavidin–biotin amplification: biotin-conjugated donkey antirabbit (1:500; Jackson ImmunoResearch Laboratory) followed by peroxidase-conjugated streptavidin (Vectastain ABC kit, Vector laboratories, Burlingame, CA, USA) or DyLight 550-conjugated streptavidin (1:200, Abcam). Lastly, fluorescently labelled sections were stained with 4′, 6-diamidino-2-phenylindole (DAPI, 1:5000, Sigma-Aldrich, St. Louis, MO, USA) to enable the visualisation of total cells. All fluorescent images were captured using a Zeiss Axio Observer Z1 epifluorescence or Zeiss confocal microscope system (Oberkochen, Germany). Bright-field images were obtained using a Leica DM6000 upright microscope (Wetzlar, Germany).

GFP expression was used to delineate the graft boundaries and estimate the volume of the graft core (delineation of the volume containing GFP+ cell bodies) and volume of innervation. Quantification of percentage of NeuN+/DAPI, density of NeuN+, percentage of Ctip2+/NeuN+ cells within the delineated GFP+ graft was acquired from images at 40× magnification. Host-derived GFAP+ density (assessed as % immunoreactive pixels) and quantification of GFP fiber density (assessed as % immunoreactive pixels) were assessed at the graft–host border, as previously described and analysed [17]. Images were captured at 40× magnification.

### 4.10. Statistical Analysis

All data are presented as mean ± standard error of the mean (SEM). Data were analysed using GraphPad Prism 6.0 software (GraphPad, San Diego, CA, USA) by one-way analysis of variance (ANOVA) with Turkey’s post hoc tests. Differences at *p* < 0.05–0.01 were considered to be statistically significant.

## Data Availability

Data are contained within the article.

## Supplementary Materials

The following supporting information can be downloaded at: https://www.mdpi.com/article/10.3390/gels8040224/s1, Figure S1: Representative photomicrographs staining with GFP+, βIII-tubulin+, DAPI+ and merged images in Cells group, Cells + sSDF group, and Cells + SAP-shSDF group, demonstrating immature neurons in all groups.
