# Supplementary material for: A Hydrogel as a Bespoke Delivery Platform for Stromal Cell-Derived Factor-1
Source: Gels. 2022 Apr 6;8(4):224. doi: 10.3390/gels8040224 (PMC9025061; doi:10.3390/gels8040224)
Supplement: Supplementary file 1 [file gels-08-00224-s001.zip › gels-1649827-supplementary.pdf]

gels

Article

A Hydrogel as a Bespoke Delivery Platform for Stromal Cell-Derived Factor-1

Yi Wang [1,2], Vanessa Penna [3], Richard J. Williams [4], Clare L. Parish [3,†] and David R. Nisbet [1,5,6,7,*,†]

1 The Graeme Clark Institute, The University of Melbourne, Melbourne, Australia; yi.wang24@unimelb.edu.au
2 Department of Biomedical Engineering, Faculty of Engineering and Information Technology, The University of Melbourne, Melbourne, Australia
3 The Florey Institute of Neuroscience and Mental Health, The University of Melbourne, Parkville, VIC 3052, Australia; vanessa.penna@florey.edu.au (V.P.); clare.parish@florey.edu.au (C.L.P.)
4 Institute for Mental and Physical Health and Clinical Translation, School of Medicine, Deakin University, Warun Ponds, VIC 3216, Australia; richard.williams@deakin.edu.au
5 Laboratory of Advanced Biomaterials, Research School of Chemistry and the John Curtin School of Medical Research, The Australian National University, Canberra 2601, Australia
6 Melbourne Medical School, Faculty of Medicine, Dentistry and Health Science, The University of Melbourne, Melbourne, Australia
7 Department of Biomedical Engineering, Faculty of Engineering and Information Technology, The University of Melbourne, Melbourne, Australia
* Correspondence: david.nisbet@unimelb.edu.au
† These authors contributed equally to this work.

Supporting information

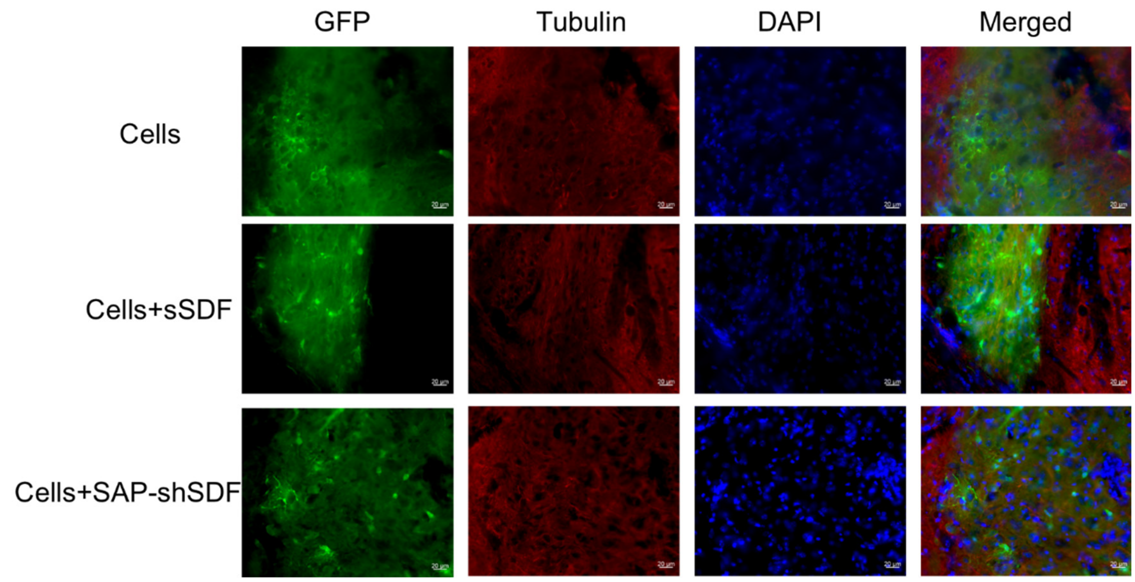

Figure S1. Representative photomicrographs staining with GFP+, βIII-tubulin+, DAPI+ and merged images in Cells group, Cells + sSDF group, and Cells + SAP-shSDF group, demonstrating immature neurons in all groups.
